# Demographic, psychosocial, and genetic risk associated with smokeless tobacco use among Mexican heritage youth

**DOI:** 10.1186/s12881-015-0188-8

**Published:** 2015-06-26

**Authors:** Anna V. Wilkinson, Laura M. Koehly, Elizabeth A. Vandewater, Robert K. Yu, Susan P. Fisher-Hoch, Alexander V. Prokhorov, Harold W. Kohl, Margaret R. Spitz, Sanjay Shete

**Affiliations:** Michael & Susan Dell Center for Healthy Living & The University of Texas School of Public Health, Austin Regional Campus, 1616 Guadalupe St., Suite 6.300, Austin, TX 78701 USA; Social and Behavioral Division, National Human Genome Research Institute, Bethesda, MD USA; Division of Cancer Prevention and Population Sciences, The University of Texas MD Anderson Cancer Center, Houston, TX USA; The University of Texas School of Public Health Brownsville Regional Campus, Brownsville, TX USA; Department of Kinesiology and Health Education, University of Texas at Austin, Austin, TX USA; Baylor College of Medicine, Houston, TX USA

## Abstract

**Background:**

Despite well-established negative health consequences of smokeless tobacco use (STU), the number and variety of alternative non-combustible tobacco products on the market have increased tremendously over the last 10 years, as has the market share of these products relative to cigarettes. While STU among non-Hispanic white youth has decreased over the last 10 years, the prevalence has remained constant among Hispanic youth. Here we examine demographic, psychosocial, and genetic risk associated with STU among Mexican heritage youth.

**Methods:**

Participants (50.5 % girls) reported on psychosocial risk factors in 2008–09 (*n* = 1,087, mean age = 14.3 years), and smokeless tobacco use in 2010–11 (mean age = 16.7 years). Participants provided a saliva sample that was genotyped for genes in the dopamine, serotonin and opioid pathways.

**Results:**

Overall 62 (5.7 %) participants reported lifetime STU. We identified five single nucleotide polymorphisms that increased the risk for lifetime use. Specifically, rs2023902 on SERGEF (OR = 1.93; 95 % CI: 1.05-3.53), rs16941667 on ALDH2 (OR = 3.14; 95 % CI: 1.65-5.94), and rs17721739 on TPH1 (OR = 1.71; 95 % CI: 1.00-2.91) in the dopamine pathway, rs514912 on TRH-DE (OR = 1.84; 95 % CI: 1.25-2.71) in the serotonin pathway, and rs42451417 on the serotonin transporter gene, SLC6A4 (OR = 3.53; 95 % CI: 1.56-7.97). After controlling for genetic risk, being male (OR = 1.86; 95 % CI: 1.02-3.41), obesity status (OR = 2.22; 95 % CI: 1.21-4.09), and both higher levels of anxiety (OR = 1.04; 95 % CI: 1.01-1.08) and social disinhibition (OR = 1.26; 95 % CI: 1.07-1.48) were associated with increased use. High subjective social status (OR = 0.78; 95 % CI: 0.64-0.93) was protective against use, while higher parental education (OR = 2.01; 95 % CI: 1.03-3.93) was associated with increased use.

**Conclusions:**

These data suggest that use of genetic risk, along with psychosocial, demographic, and behavioral risk factors may increase our ability to identify youth at increased risk for STU, which in turn may improve our ability to effectively target prevention messages to Mexican heritage youth.

## Background

The negative health consequences of smokeless tobacco use are well documented [1–3]. Smokeless tobacco use (STU) is associated with increased risk for head and neck cancers [1], and among women, poor birth outcomes [2]. Maternal smokeless tobacco use increases rates of stillbirth, low birth weight and alters the male-to-female live birth ratio [3]. This is particularly concerning given the number and variety of alternative tobacco products on the market have increased tremendously over the last 10 years, as has the market share of these products relative to cigarettes [4].

Although the number of youth using chewing tobacco, snuff and/or dipping tobacco decreased in the United States (US) at the national level between 1995 and 2003, evidence suggests use has subsequently stabilized [5]. In 2013, an estimated 8.8 % of youth in grades 9 through 12 in the US reported use of chew, snuff or dip during the 30 days before the survey [5]. Of importance, between 2002 to 2011 reported poly-tobacco product use (e.g. cigarettes, smokeless tobacco products, e-cigarettes) has increased among people under the age 26, even while poly-tobacco product use in the overall population has decreased. This increase is largely attributable to increases in smokeless tobacco product use [6]. In Texas, the overall smokeless tobacco prevalence in 2013 was similar to the national prevalence, as 8.1 % of youth in grades 9 through 12 reported use of chew, snuff or dip during the 30 days before the survey [5]. Although smokeless tobacco use (STU) among non-Hispanic white youth has decreased over the last 10 years, the prevalence of use has remained constant among Hispanic youth [5].

Given that Hispanics are a growing ethnic minority group throughout the US, these trends are alarming and suggest that a better understanding of the risk factors associated with smokeless tobacco use among young Hispanics is both timely and warranted to guide the development of culturally appropriate prevention programs. Thus, the purpose of this paper is to examine demographic, psychosocial, and genetic risk associated with the use of smokeless tobacco products among a cohort of Mexican heritage youth, the largest sub-group of Hispanics in the US, residing in Houston, Texas. Given the trends in poly-tobacco use [6], we examined whether psychosocial correlates associated with cigarette experimentation among the youth in this cohort [7–9] would also correlate with use of smokeless tobacco products. A better understanding of the similarity and differences in risk factors for different tobacco products will facilitate the development of interventions designed to prevent their use.

## Methods

Participants in the current analysis were recruited in 2005–06, from an on-going population-based cohort of Mexican American households established by the Department of Epidemiology at The University of Texas M.D. Anderson Cancer Center in July 2001, known as the Mexican American Cohort Study (MACS). Initial recruitment into the MACS took place through probability random digit dialing, door-to-door recruitment, intercepts, and networking. A detailed description of the MACS data collection has been published elsewhere [10].

A total of 3,000 MACS households, with age-eligible adolescents between the ages of 11 and 13 years were identified for recruitment into an adolescent cohort. This nested adolescent cohort, known as the Mexican American Tobacco Use in Children (MATCh) cohort, was established to examine both genetic and non-genetic determinants of tobacco use initiation. Of the first 1,425 potential households contacted, just over 90 % agreed to allow their child to participate in the study (*n* = 1,328). In households with two or more eligible children, the child with the most recent birthday was selected for enrollment into the study. After obtaining informed consent and assent, survey data were collected via personal interviews that took place in the family home. In addition, at the end of the baseline interview, all participants provided a saliva sample to be used for genotyping. After a brief personal interview in which acculturation and demographic data were collected, the adolescents were given a personal digital assistant (PDA) to complete a survey in order to assure confidentiality of their responses from their parents. Participants received a $25 gift certificate to compensate for their time. A detailed description of data collection procedures has been published [11]. The Institutional Review Board at UT MD Anderson approved all aspects of this study.

Three waves of data were collected from the MATCh cohort. Of the 1,328 participants who enrolled in the study at baseline in 2005–06 (wave 1), 1,154 (87 %) participated in 2008–09 (wave 2), and 1,000 (75 %) participated in 2010–11 (wave 3). The risk factor data presented in the current analysis were gathered at wave 2 in 2008–09, while the outcome data were gathered through wave 3 in 2010–11.

### Measures

#### Smokeless tobacco use

The main outcome of interest, self-reported lifetime smokeless tobacco use (STU), was coded as a binary variable based on responses to the question “How many times in your life have you used chewing tobacco or snuff?”, asked at each data collection wave. Participants who reported that they had never tried smokeless tobacco at any data collection wave were coded as “0” and called “never tried,” and those who reported having used chewing tobacco or snuff at least once, on any data collection wave, were coded as “1” and referred to as “ever tried.” Thus this variable reflects lifetime or prevalent, although not necessarily current STU.

#### Covariates

Based on the increase in poly-tobacco use [6], several demographic and psychosocial risk factors associated with cigarette smoking among adolescents in this cohort [7–9] were examined as potential covariates of STU. The demographic variables included were age (examined as a continuous variable), socioeconomic status (SES) [12], and gender [13, 14]. Parental education attainment was used as a proxy for SES and was divided into three categories: less than high school, high school graduate, and more than high school, with less than high school serving as the reference category. Female sex served as the reference category for gender. We also examined body mass index (BMI), which was calculated using the height and weight measurements taken by trained interviewers at each interview, because it is associated with smoking behavior among youth in this cohort [7]. SECA scales and stadiometers were used to gather these data following a standard protocol [7]. Three weight categories were created using standard age-gender specific growth curves [15]. Due to the small number of participants with a BMI below the 5^th^ percentile, the underweight and normal weight groups were collapsed in to one group.

Psychosocial covariates examined included anxiety [7], sensation seeking [8], and subjective social status [9]. We assessed anxiety using Spielberger’s trait anxiety scale [16]. The scale has 20 items that assess trait anxiety, with response options ranging from “not at all” to “very much so” on a 4-point scale. The scale has been validated in US Spanish-speaking samples [17, 18] and has been shown to have good reliability based on data from our participants (Cronbach’s alpha = 0.86). Participant anxiety scores reflect a composite of all item scores. Social disinhibition, one aspect of sensation seeking was assessed using a seven-item subscale from the Sensation Seeking Scale for Children [19]. Participants were asked to choose the option on each question that most describes how they feel, for instance “a) I don’t do anything I think I might get in trouble for” or “b) I like to do new and exciting things, even if I think I might get in trouble for doing them.” Based on our participants, the scale demonstrates acceptable reliability (Cronbach’s alpha = 0.68). Subjective social status was measured using the MacArthur Scale of Subjective Social Status adapted for adolescents [20]. Higher scores on the three scales reflect higher levels of anxiety, social disinhibition, and perceived subjective social status, respectively.

#### Single Nucleotide Polymorphism (SNP) selection and genotyping

Candidate genes were identified from published reviews [21] and PubMed searches using the following key words: sensation seeking, novelty seeking, risk taking, gambling, smoking, and alcohol use. This list was cross-referenced with the Gene Ontology Database (http://pid.nci.nih.gov/) and Kegg Pathway to confirm pathway information. Tagging SNPs were selected from the International HapMap Project (Release 21 with NCBI build 36; http://www.hapmap.org). The following selection criteria were used: located in the respective gene or within 10 kb upstream or downstream of the gene ends to cover the regulatory regions; minor allele frequency (MAF) >5 % in various ethnic groups; and not already represented by a current tag SNP at a linkage disequilibrium (LD) of *r*^*2*^ > 0.80. We also included SNPs in coding (synonymous SNPs, nonsynonymous SNPs) and regulatory regions (promoter, splicing site, 5-UTR, and 3-UTR). Following this approach, a total of 672 SNPs on 58 candidate genes were identified and examined in the current study. Genotyping was conducted following standard procedures, which are described in detail elsewhere [22].

### Statistical analysis

Pearson’s chi-square tests were conducted to examine the associations between lifetime STU and the categorical covariates, adolescent gender, parental educational attainment, and adolescent BMI defined weight category. Student’s t-tests were used to examine mean differences in age, anxiety, subjective social status and social disinhibition, by STU status (Table 1). Additional chi-square tests were conducted to compare genotypes by STU (Table 2, panel A). For some individuals, outcome or covariate data were missing. We performed complete case analyses, a standard analytical approach. Also, given that the analyses are exploratory in nature, we did not correct for multiple comparisons in the current analysis [23].

**Table 1:** Demographic characteristics and psychosocial constructs by lifetime smokeless tobacco use (*N* = 1,087)

	Smokeless tobacco use		
	Never	Ever	
	N (%)	N (%)	*p*-value
Gender			0.003
Male	496 (92.2)	42 (7.8)	
Female	529 (96.4)	20 (3.6)	
Parental education			0.123
Less than HS	672 (94.5)	39 (5.5)	
HS	181 (96.3)	7 (3.7)	
More than HS	172 (91.5)	16 (8.5)	
Body Mass Index			0.002
Underweight/Normal	523 (95.8)	23 (4.2)	
Overweight	193 (96.5)	7 (3.5)	
Obese	309 (90.6)	32 (9.4)	
	M (SD)	M (SD)	*p*-value
Age at survey	14.3 (1.4)	14.6 (1.2)	0.029
Anxiety	37.9 (9.2)	42.7 (9.6)	<0.001
Subjective Social Status	7.8 (1.4)	6.9 (1.9)	<0.001
Social Disinhibition	3.2 (1.9)	4.2 (1.6)	<0.001

**Table 2:** Distribution of gene variants retained after the manual backward elimination for lifetime smokeless tobacco use (Panel A), and comparison of gene variants retained after the manual backward elimination between HapMap and MATCh samples (Panel B)

		Panel A			Panel B	
		Smokeless Tobacco Use			HapMap	MATCh
Genes	Dominant	Never	Ever		minor allele	minor allele
	Model	(*N* = 1,025)	(*N* = 62)			
	N (%)	N (%)	N (%)	*p*-value	%	%
SERGEF (rs2023902)				0.005		
0	856 (78.7)	816 (79.6)	40 (64.5)		0.120	0.108
1	209 (21.3)	209 (20.4)	22 (35.5)			
TPH1 (rs17721739)				0.002		
0	811 (74.6)	775 (75.6)	34 (54.8)		--	0.130
1	276 (25.4)	250 (24.4)	28 (45.2)			
TRH-DE (rs514912)				<0.001		
0	428 (39.4)	419 (40.9)	9 (14.5)		0.360	0.386
1	659 (60.6)	606 (59.1)	53 (85.5)			
ALDH2 (rs16941667)				0.002		
0	970 (89.2)	922 (90.0)	48 (77.4)		0.050	0.057
1	1,117 (10.8)	103 (10.0)	14 (22.6)			
SCL6A4 (rs4251417)				0.001		
0	1,017 (93.6)	965 (94.1)	52 (83.9)		0.030	0.034
1	70 (6.4)	60 (5.9)	10 (14.3)			

For each SNP, allelic data were recoded into two potential genetic models: additive and dominant. Two separate logistic regression analyses were then conducted for each candidate SNP (one for each genetic model), controlling for age and gender. Principal components-based clustering analysis was also conducted to test for possible underlying ethnic stratification [24–26], and was performed using PLINK v.1.07 [27]. The goal of the analyses is to cluster samples in subsets containing at least one STU and one non-STU based on pairwise population concordance test. We applied this approach to identify clusters for each individual. We then used these clusters as covariates in logistic regression analyses.

The best-fitting genetic models (i.e. dominant or additive) for each SNP with significant regression results were examined simultaneously in a multiple logistic regression model, which also included demographic and psychosocial risk factors, BMI, and clusters to adjust for underlying ethnic stratification. A final model of significant SNPs, adjusting for the demographic and psychosocial variables, was determined using a manual backwards elimination process such that those SNPs with a p-value >0.05 in the multivariable model were removed (Table 2, panel B).

## Results

DNA from 1,265 participants was available for analysis. Of the 1,154 who completed wave 2, 43 participants were missing data on BMI and 24 on parental education. The final sample size available for the analysis based on wave 2 was 1,087. At wave 1, 22 participants reported lifetime STU, by wave 2 in 2008–09, 45 participants reported lifetime STU, and by wave 3, 5.7 % or 62 of the 1,087 participants included in the current analysis reported lifetime STU. We examined differences in gender distribution, mean age, parental educational attainment distribution, mean subjective social status, mean body mass index, and mean anxiety between participants with complete data and included in the analysis and those with missing data and excluded from the analysis. Participants with missing data and who were excluded from the analysis were comparable on all baseline parameters save one. They were significantly older than those included in the analysis (*p* < 0.01).

Bivariate associations between STU status and the above mentioned demographic and psychosocial variables are shown in Table 1. A higher percentage of males reported STU than females (*p* = 0.003) and a higher percentage of obese youth reported STU than their overweight, normal weight, and underweight counterparts (*p* = 0.002). Although not significant, a higher percentage of youth whose parents had more than a high school education reported STU than youth whose parents completed less education. Ever users of smokeless tobacco reported higher levels of anxiety (*p* < 0.001), lower subjective social status than never users (*p* < 0.005), and higher levels of social disinhibition than never users (*p* < 0.001).

The five SNPs identified through the manual backwards elimination process are presented in Table 2. Specifically, we identified rs2023902 on secretion regulating guanine nucleotide exchange factor gene in the dopamine pathway, rs17721739 on tryptophan hydroxylase 1 gene in the dopamine pathway, rs514912on thyrotropin-releasing hormone degrading enzyme gene in the serotonin pathway, rs16941667 on aldehyde dehydrogenase 2 gene in the dopamine pathway, and rs42451417 on the serotonin transporter gene, SLC6A4For each SNP, a higher proportion of ever users carried the minor allele compared to never users. For each SNP, a higher proportion of ever users carried the minor allele compared to never users. As can be seen in Table 2 (Panel B), the minor allele frequency in our data is similar to that reported by the HAPMAP project based on a sample of 100 Mexicans.

The results from the multivariate analyses are presented in Table 3. Male gender and having a parent with more than a high school education were associated with a roughly 2-fold increased odds of lifetime STU, while age at survey was not significant. Obesity status was associated with increased odds of ever use (OR = 2.22; 95 % CI: 1.21-4.09). Anxiety was associated with increased odds of lifetime STU (OR = 1.04; 95 % CI: 1.01-1.08). Higher levels of subjective social status conferred a protective influence on ever use of smokeless tobacco (OR = 0.78; 95 % CI: 0.64-0.93). Social disinhibition was significantly associated with increased odds of lifetime STU (OR = 1.26; 95 % CI: 1.07-1.48). Carrying the minor allele of each SNP was associated with an increase in odds for STU. For example, rs17721739 on TPH1 was associated with a 71 % (1.00-2.91) increase in odds of use, whereas rs4251417 on SCL6A4 was associated with over a 3-fold increase in odds of use (OR = 3.52; 95 % CI: 1.56-7.97).

**Table 3:** Adjusted odds ratios* (OR) and 95 % confidence intervals (CI) for lifetime STU by demographic, psychosocial and genetic risk factors (*N* = 1,087)

	OR	95 % CI	*p*-value
Demographics			
Male sex	1.86	1.02-3.41	0.044
Age at survey	1.15	0.87-1.53	0.324
Parental Education (< HS)			
HS	0.87	0.36-2.07	0.748
> HS	2.01	1.03-3.93	0.040
Body Mass Index (Normal)			
Overweight	0.57	0.28-1.47	0.248
Obese	2.22	1.21-4.09	0.010
Psychosocial			
Anxiety	1.04	1.01-1.08	0.012
Social Disinhibition	1.26	1.07-1.48	0.006
Subjective Social Status	0.78	0.64-0.93	0.007
SNPs			
SERGEF (rs2023902)	1.93	1.05-3.53	0.033
TPH1 (rs17721739)	1.71	1.00-2.91	0.048
TRH-DE (rs514912)	1.84	1.25-2.71	0.002
ALDH2 (rs16941667)	3.14	1.65-5.94	<0.001
SCL6A4 (rs4251417)	3.53	1.56-7.97	0.002

*Adjusted for all variables in the model and population stratification

## Discussion

To the best of our knowledge, this is the first study to examine genetic and non-genetic risk factors associated with STU in an adolescent-age sample of Mexican heritage youth. We found five SNPs with a positive association with ever use of smokeless tobacco. The odds of lifetime STU were higher at higher numbers of risk SNPs. Seventeen percent of the youth with three or more risk alleles reported lifetime STU compared to two percent with no risk alleles. Thus, these results indicate that genetic variants play an important role in susceptibility to STU.

We also examined several non-genetic correlates of cigarette use [7–9] to examine whether they demonstrate similar associations with STU. After controlling for the genetic risk, being male and obese, as well as reporting higher levels of anxiety and social disinhibition were associated with increased odds of STU. Higher subjective social status was protective against STU yet having parents with more than a high school education increased the odds of STU.

Chewing tobacco is predominantly a male behavior [28] and in particular among Mexican American youth [29]. Consistent with previous research, we found males were two times more likely to use smokeless tobacco than females. Youth whose parents reported more than a high school education were also at higher risk for STU than those with less education. While all tobacco use is inversely associated with SES in many populations [12], our finding is consistent with recent data from Hispanic youth [28], which demonstrate a positive association between SES and tobacco use. Given the overall low level of educational attainment among the participants’ parents, these results suggest that a certain level of disposable income is an important risk factor for STU among Mexican heritage youth during early to mid-adolescence.

In the current analysis, higher BMI scores and obese weight status were associated with ever STU. This result is consistent with previous research conducted among young US military recruits, among whom STU was also associated with increased BMI [30]. Of note, while STU was associated with increased BMI in the current analysis, in a previous analysis based on the same cohort, we found that higher BMI offered a protective effect against experimenting with cigarettes [7]. Together these results suggest that STU may not offer the same perceived weight control benefits that many youth associate with smoking cigarettes [31, 32].

Evidence suggests that anxiety is a risk factor for cigarette use [33] and that regular cigarette smoking is associated with anxiety disorders [34]. Although several studies have reported no association between anxiety and STU [35, 36], in our analysis, we found that higher levels of anxiety were associated with STU at each home assessment. When we consider the significant association between obesity status and STU in conjunction with higher anxiety as a risk factor, our results are consistent with Botvin et al. [37] who reported that eating as a coping response to anxiety provoking events increases the risk for STU.

Although several studies have reported a protective effect of subjective social status on cigarette use [9, 38, 39], this is the first to report a similar protective effect against STU. The effect was found after controlling for more objective measures of status, specifically parental educational attainment. This suggests that among the participants in our study, subjective evaluations of status are associated with STU net the influence of more objective measures of status.

In the current analysis, social disinhibition, a self-reported aspect of sensation seeking, was associated with STU during mid-adolescence. Consistent with our finding, a recent study found that sensation seeking tendencies were associated with an interest in trying dissolvable tobacco products [40].

### Genetic polymorphisms and smokeless tobacco use

Our results suggest that genetic polymorphisms are associated with STU among Mexican heritage youth. We examined genetic variants associated with sensation seeking behaviors and found that variants on several genes served to increase risk of chewing tobacco. Specifically, participants with at least one copy of the minor allele for SNPS in SLC6A4 (rs4251417), TPH1 (rs17721739), ALDH2 (rs16941667) TRH-DE (rs514912), and SERGEF (rs2023902) were associated with increased likelihood of lifetime STU. To the best of our knowledge, this is the first report of an association between lifetime STU and each of these five SNPs that demonstrated a significant increase in risk.

Although the genes we examined in the current study were selected based on existing evidence demonstrating a relationship with sensation seeking behaviors or self-reported sensation seeking tendencies, four of the genes that demonstrated a significant association with chewing tobacco, are also associated with anxiety. For example, the s-variant of the serotonin transporter gene, SLC6A4, is associated with anxiety and depression [41] and the SNP we identified (rs4251417) has been implicated in the etiology of depression and anxiety disorders [42]. Mutations on TPH1 are associated with an elevated risk for a variety of diseases and disorders, including, somatic anxiety [43], suicidal behavior [44], and nicotine addiction [45]. The ALDH2 genotype demonstrates a strong association with alcohol dependence among individuals with a comorbid anxiety disorder [46]. The SNP we identified on ALDH2 (rs16941667) is associated with increased risk for gastric cancers [47]. Although research on TRH-DE, which is responsible for deactivating the release of the thyrotropin-releasing hormone (TRH) in humans [48], is sparse, a recent study in rats supports the participation of TRH during response to anxiety provoking situations [49]. The remaining SNP we identified on the SERGEF gene, also known as DELGEF, is associated with Usher 1C syndrome [50], a hereditary syndrome in which children are born with severe hearing and visual impairments, and many experience severe balance problems.

Taken together, our results suggest that anxiety may be directly linked to STU in youth of Mexican heritage. During adolescence, when the habit is forming, we found higher BMI and higher anxiety were associated with STU at all three time points, while others have reported STU has been associated with eating in response to anxiety provoking situations [37]. Together these results underscore the possibility that youth may try smokeless tobacco products, such as chewing tobacco, as a means to cope with anxiety, rather than as an expression of sensation seeking tendencies. Consistent with this hypothesis, nicotine chewing gum has been used successful to treat obsessive compulsive disorder [51, 52], further underscoring the possibility that anxious youth use these products to cope with anxiety.

### Strengths & limitations

Our study has methodological strengths. First, our sample came from a population-based cohort, representing an ethnically homogenous and predominantly low-income population of Mexican origin youth. This population is largely understudied and underserved. Second, given the sensitive nature of some of the questions, using PDAs, which ensured that participants read and answered the questions without concern for their parents hearing or seeing their responses, is a strength of our study.

Our study has some limitations. The main limitation of our analysis is the lack of an independent replication sample, which is fairly common among minority populations; thus our findings ought to be considered as preliminary and exploratory. Second, participants in our sample were all of Mexican origin. The homogeneity of our sample hinders the generalizability of our conclusions to other populations, including other Hispanic youth from different countries of origin. A third limitation pertains to the relatively small number of individuals who reported lifetime STU implying limited statistical power for this preliminary study. The data were assessed via self-report and unverified using biological samples or other methods of cross-validation; STU may have been underreported by some and was inconsistently reported by roughly 20 %. Evidence suggests, however, that the validity of self-reported data tends to increase when participants believe biological samples are requested, which was the case in our study [53]. Thus consistent with tobacco research in adolescents, all reports of yes were counted as a yes irrespective of subsequent self-reported changes in status; such an approach enabled us to use the maximum number of smokeless tobacco users.

## Conclusions & implications

We found male gender, obesity status, lower subjective social status, and higher self-reported social disinhibition and anxiety level, along with five SNPs, four of which have previously been associated with anxiety, were associated with STU. Overall, among Mexican heritage youth, if replicated, our results suggest that using genetic risk, along with psychosocial, demographic, and behavioral risk factors to identify youth at high risk for STU may improve our ability to effectively target prevention messages to high-risk youth. In turn this may improve the efficacy of targeted intervention strategies to prevent STU. Our results underscore the possibility that experimentation with smokeless tobacco may reflect a way to cope with anxiety. Therefore addressing issues related to anxiety in primary prevention efforts aimed to reduce experimentation with smokeless tobacco may enhance the efficacy of the intervention. To the best of our knowledge, these are among the first results to simultaneously report on psychosocial, demographic, and genetic risk associated with STU, and therefore they must be viewed as preliminary.
